# Genotyping and subtyping *Cryptosporidium parvum* and *Giardia duodenalis* carried by flies on dairy farms in Henan, China

**DOI:** 10.1186/1756-3305-7-190

**Published:** 2014-04-17

**Authors:** Zifang Zhao, Haiju Dong, Rongjun Wang, Wei Zhao, Gongyi Chen, Shouyi Li, Meng Qi, Sumei Zhang, Fuchun Jian, Jinfeng Zhao, Longxian Zhang, Haiyan Wang, Aiqin Liu

**Affiliations:** 1 College of Animal Science and Veterinary Medicine, Henan Agricultural University, Zhengzhou 450002, PR China; 2 International Joint Research Laboratory for Zoonotic Diseases of Henan, Henan Agricultural University, Zhengzhou 450002, PR China; 3 Department of Parasitology, Harbin Medical University, Harbin 150081, PR China; 4 Department of Animal Science, Henan Vocational College of Agriculture, Zhongmu 451450, Henan Province, PR China

**Keywords:** *Cryptosporidium*, *Giardia duodenalis*, Fly, Genotyping, Subtyping

## Abstract

**Background:**

*Cryptosporidium* and *Giardia* are important causes of diarrhea diseases in humans and animals worldwide, and both of them are transmitted by the fecal–oral route, either by direct contact or by the ingestion of contaminated food or water. The role of flies in the mechanical transmission of *Cryptosporidium* and *Giardia* has been receiving increasing attention. To date, no information is available in China about the occurrence of *Cryptosporidium* and *Giardia* in flies. We here investigated *Cryptosporidium* and *Giardia* in flies on dairy farms in Henan Province, China, at the genotype and subtype levels.

**Methods:**

Eight hundred flies were randomly collected from two dairy farms from July 2010 to September 2010 and were divided evenly into 40 batches. The fly samples were screened for the presence of *Cryptosporidium* and *Giardia* with nested PCR. *Cryptosporidium* was genotyped and subtyped by analyzing the DNA sequences of small subunit rRNA (SSU rRNA) and 60-kDa glycoprotein (*gp60*) genes, respectively. The identity of *Giardia* was determined by sequence analyzing of the triosephosphate isomerase (*tpi*), glutamate dehydrogenase (*gdh*), and β-giardin (*bg*) genes.

**Results:**

Forty batches of flies had 10% of contamination with *Cryptosporidium* or *Giardia*, with a mixed infection of the two parasites in one batch of flies. The *Cryptosporidium* isolates were identified as *C. parvum* at the SSU rRNA locus, and all belonged to subtype IIdA19G1 at the *gp60* locus. The *Giardia* isolates were all identified as assemblage E of *G. duodenalis* at the *tpi*, *gdh*, and *bg* loci. One novel subtype of assemblage E was identified based on the *gdh* and *bg* loci.

**Conclusions:**

This is the first molecular study of *Cryptosporidium* and *Giardia* in flies identified at both genotype and subtype levels. SSU rRNA and *gp60* sequences of *C. parvum* in flies was 100% homologous with those derived from humans, suggesting flies act as an epidemiological vector of zoonotic cryptosporidiosis. The variable PCR efficiencies observed in the analysis of *Giardia* at different loci suggest that we should use the multilocus genotyping tool in future studies to increase the detection rate, and importantly, to obtain more complete genetic information on *Giardia* isolates.

## Background

*Cryptosporidium* and *Giardia* are commonly regarded as zoonotic parasites responsible for diarrheal diseases in humans worldwide. The diarrhea caused by cryptosporidiosis may become profuse and chronic, depending on the health status of the host, and appears to be life-threatening in people with various immune-system deficiencies. Many studies have confirmed the high mortality associated with *Cryptosporidium* infection in patients with human immunodeficiency virus infection [1-4]. A single *Cryptosporidium* oocyst has been reported to initiate infection in immunosuppressed persons. Similarly, the pathogen also debilitates immunocompetent persons in whom the disease can be caused by as few as 10 oocysts [5]. The 50% infectious dose (ID_50_) of geographically diverse isolates of *C. parvum* for immunocompetent people ranges from 9 to 1,042 oocysts [6]. The pathogenicity of *Giardia* is generally considered to be less severe in humans than that of *Cryptosporidium*, but the parasite can lead to growth and developmental retardation in children through malnutrition, even in asymptomatic cases [7].

The broad zoonotic aspects of these diseases complicate the epidemiology of both cryptosporidiosis and giardiasis because both occur in a wide range of reservoir hosts. Both these parasites can persist for long periods of time in the environment (i.e., in water, soil, on vegetation and other food resources) and maintain their infectivity even under harsh environmental conditions [8]. People acquire cryptosporidiosis or giardiasis by digesting oocysts or cysts excreted from infected hosts, respectively, mainly through either direct host-to-host contact or indirectly by the consumption of contaminated water or food [9,10]. Water- and food-borne cases and outbreaks of cryptosporidiosis and giardiasis have been extensively documented in several countries and regions [11-22]. However, in analyses of the correlative risk factors for the two parasitic diseases, especially during food-borne outbreak events involving *Cryptosporidium* oocysts and *Giardia* cysts, flies have drawn attention because of their role in the mechanical transmission of these food-borne infections.

When researchers consider the vehicles that transport these parasites between feces and food, flies are of greater concern than other arthropods (such as cockroaches and dung beetles) because of the huge numbers of flies in the environment, especially in locations where contaminated human and animal feces are readily accessible to them, such as in rural livestock-raising areas. Outbreaks of food-borne diarrheal diseases display distinct seasonal patterns, which coincide with seasonal increases in the abundance of flies during the warm months of the year [23]. The control of flies is closely related to declines in the numbers of outbreaks and human cases of food-borne diseases [24]. Several studies have confirmed that the fly is involved in the mechanical transmission of *C. parvum* and *G. duodenalis* in a variety of settings, and have demonstrated that flies can act as mechanical vectors [23,25-35]. Flies can carry *Cryptosporidium* oocysts and *Giardia* cysts on their body surfaces (exoskeletons) and in their digestive tracts because of their vagility and feeding mechanisms [23,25,29,35]. Flies are also reported to be able to carry 4–131 *Cryptosporidium* oocysts each for at least three weeks, and the oocysts deposited by them have been shown to be infectious in mice [34]. Although *G. duodenalis* cysts have been reported in flies, their viability and infectivity have not been determined.

Cattle manure is a favorite breeding place, food source, and landing site for filth flies. It has been reported that cattle barns are sites at which house flies can reproduce throughout the winter or can overwinter [34]. In China, currently there are no reports of flies carrying *Cryptosporidium* or *Giardia*. The aims of the present study were to determine the occurrence of *Giardia* cysts and *Cryptosporidium* oocysts in flies captured on dairy farms, and to assess the role of flies as vectors in the transmission of cryptosporidiosis and giardiasis by genotyping and subtyping tools.

## Methods

### Sites and methods of collecting flies

The flies analyzed in the present study were captured on two dairy farms in Zhengzhou City, Henan Province, where all preweaned calves were kept and where livestock infecting with *Cryptosporidium* and *Giardia* are known to occur. Collection began on 10 July 2010 and lasted until 25 September 2010; the average air temperature was 27-32°C and the relative humidity was 35%-90%. Briefly, four fly-catching papers (Dahao Daily Chemicals Co., Ltd., Guangdong, China) were set at different locations in each barn. The flies were taken from the papers with forceps at 3-4-day intervals [34]. Eight hundred of the flies were randomly selected, counted, and divided into batches of 20. Among which, more than 90% of files belonged to *Musca domestica*, and only a small number of them were *Chrysomya megacephala* and *Sarcophaga* sp. Each batch was resuspended separately in a 10-ml tube in 5 ml of phosphate-buffered saline, transported to the laboratory in a cooler with ice packs, and stored at 4°C until processing.

### Processing flies for oocysts/cysts

The 40 batches of flies were each ground in a mortar until crushed so that the parasites they carried internally were released into the homogenates [29]. The homogenates were then immediately filtered with a 198 μm sieve, and the filtrates were centrifuged at 1500× g for 5 min at room temperature. This ensured the recovery of the particles derived from the exoskeletons and digestive tracts of the flies in the sedimented debris.

### DNA extraction

The genomic DNA of *Cryptosporidium* and *Giardia* from the fly-derived debris was extracted from 100 mg of processed sample using the E.Z.N.A. Stool DNA Kit (Omega Biotek Inc., Norcross, GA, USA), in accordance with the manufacturer-recommended procedure. The eluted DNA (200 μl) was stored at −20°C until its analysis with PCR.

### Genotyping and subtyping *Cryptosporidium* and *Giardia*

All DNA preparations were screened for the presence of *Cryptosporidium* DNA with the nested PCR amplification of an approximately 830 bp fragment of the SSU rRNA gene, and were identified to the species/genotype level as previously described by Xiao *et al.*[36]. The *C. parvum*-positive samples were subtyped with the nested PCR amplification of an approximately 850 bp fragment of the *gp60* gene, as previously described by Alves *et al.*[37].

The *G. duodenalis* genotypes and subtypes were determined with three distinct protocols using nested PCRs targeting the *tpi, gdh*, and *bg* genes of the parasite; fragments of approximately 530 bp, 530 bp, and 510 bp, respectively, were amplified corresponding to the partial genes [38-41].

Each DNA preparation was performed three times by using 2 μl of DNA per PCR. All the nested PCR products described above were visualized by electrophoresis in 1.5% agarose gel stained with ethidium bromide before sequencing.

### DNA sequence analysis

All secondary PCR products were sequenced using the secondary PCR primers on an ABI PRISM™ 3730xl DNA Analyzer using the BigDye Terminator v3.1 Cycle Sequencing Kit (Applied Biosystems, Carlsbad, CA, USA). The accuracy of the sequencing data was confirmed by sequencing in both directions, and further PCR products were sequenced if necessary for some DNA preparations. The genotype and subtype identities of the *Cryptosporidium* and *G. duodenalis* isolates were established with direct comparisons, using ClustalX 1.83, of the acquired nucleotide sequences with each other and with reference sequences downloaded from GenBank.

## Results

### Distribution of *Cryptosporidium* and *Giardia* on the two dairy farms

Overall, 10% of the batches of flies were contaminated by one or other of the two parasites, with a mixed infection of them both in one sample (Table 1). However, the rates of occurrence of *Cryptosporidium* and *Giardia* in the batches of flies differed on the two farms. The occurrence of *Giardia* was higher than that of *Cryptosporidium* in the batches of flies on Farm A, with *Giardia* in 15% (3/20) of batches and *Cryptosporidium* in 10% (2/20) of batches. In contrast, more batches of flies carried *Cryptosporidium* (10%, 2/20) than carried *Giardia* (5%, 1/20) on Farm B.

**Table 1:** **Genotypes and subtypes of *G. duodenalis *** **and *C. parvum *** **in flies on two dairy farms**

**Sampling site**	**No. of flies**	**No. of batches**	***Cryptosporidium***		***Giardia duodenalis***		
			**SSU rRNA (n)**	***gp60 *** **(n)**	***tpi *** **(n)**	***gdh *** **(n)**	***bg *** **(n)**
Farm A	400	20	*C. parvum* (2)	IIdA19G1 (2)	assemblage E (2)	Assemblage E (3)	Assemblage E (3)
Farm B	400	20	*C. parvum* (2)	IIdA19G1 (2)	assemblage E (1)	Assemblage E (1)	Assemblage E (1)

### Genotyping and subtyping *Cryptosporidium*

Forty batches of flies were all screened for the presence of *Cryptosporidium* using PCR amplification of the SSU rRNA gene. Four samples (two at each farm) were positive for *Cryptosporidium* and were identified as *C. parvum*. The four *C. parvum* sequences were identical to each other and had 100% homology to previous isolates derived from cattle [GenBank: HQ009805, AB513857-AB513881, AB441687, EF611871, AF093493, and JX416362] and humans [GenBank: EU331237-EU331241, DQ523504, and DQ656352]. At the *gp60* locus, all four *C. parvum* isolates produced the expected PCR product, which was successfully sequenced. They all belonged to subtype IIdA19G1 and shared 100% homology with previous sequences of subtype IIdA19G1 derived from cattle [GenBank: HQ009809 and JX416369] and humans [GenBank: DQ280496 and JF691561].

### Genotyping and subtyping *G. duodenalis*

Three, four, and four of the 40 batches of flies were identified as assemblage E of *G. duodenalis* at the *tpi*, *gdh*, and *bg* loci, respectively. At the *tpi* locus, the three *tpi* nucleotide sequences were identical and belonged to *G. duodenalis* assemblage E (accession number KJ009339), which has been reported in cattle [GenBank: EF654686 and KC778540], sheep [GenBank: JF792416 and JF792421], and goats [GenBank: HQ283233, EU189328, EU189333-EU189340, EU189343, EU189344, EU189349, EU189351-EU189353, EU189355, and EU189356]. Novel subtypes of assemblage E were found at the *gdh* and *bg* loci. The four *gdh* and four *bg* assemblage E sequences (accession number KJ009340 for the *gdh* gene and KJ009341 for the *bg* gene) were identical to each other, but shared only 99.8% homology with the previously reported sequences of goat-derived *G. duodenalis* isolates [GenBank: AY826198 and DQ116624].

## Discussion

Human intestinal parasites are relatively common worldwide, especially in the rural areas of developing countries because of poverty, poor environmental hygiene, and inadequate health services. In Spain, the prevalence of human cryptosporidiosis in hospitals is higher in patients from rural districts (3.4%, 23/675) than in those from urban areas (1.2%, 17/1429), and these results may be related to the general presence of flies, which naturally harbor *Cryptosporidium* oocysts, in rural areas [31]. It has been confirmed in field studies and laboratory experiments that flies carry human parasites on their exoskeletons and in their digestive tracts, including *Cryptosporidium* and *Giardia*[28,42]. Because *Cryptosporidium* oocysts and *Giardia* cysts are common in the environment, flies, especially coprophagous flies, may be involved in the mechanical dissemination of the parasites via their exoskeletons, fecal deposition, and regurgitation after contact with infected materials. They then deposit these oocysts/cysts on the surfaces they visit. Filth flies are reported to have the capacity to travel up to 20 miles [29], and a positive relationship has been demonstrated between the abundance of flies at a specific site and the number of *Cryptosporidium* oocysts carried by them [27]. The transmission of human pathogens by adult flies has been reported to occur via mechanical dislodgment [28].

In this study, 40 batches of flies had 10% of infection rate for *Cryptosporidium* and *Giardia* by molecular methods. Previously, variable infection rates for both these parasites have been reported in flies conducted in five countries. The occurrence of *Cryptosporidium* in flies ranges from 1.81% to 83.3%, and of *Giardia* ranges from 3.34% to 84% (Table 2). The infection rates are complicated and are probably affected by many factors, including the collection site, sample size, sensitivity and specificity of diagnostic technique, and so on. In addition, differences in collection seasons and the sites of fly capture might also influence the infection rates of the parasites, because environmental temperature and humidity directly affect the growth and development of flies, resulting in changes in the numbers and vitality of flies. Seasonal increases in the abundance of flies during the warm months of the year are well known. Outbreaks of food-borne diarrheal diseases show seasonal fluctuations, which accord with the seasonal changes in fly numbers [23]. Flies tend to feed on and breed in filth, especially animal manure and human excrement. There is also a close relationship between the environments at the collection sites and the fly populations. A study of the potential role of flies as a transport host for *Cryptosporidium* spp. in different filth conditions, conducted in Ethiopia, found that *Musca domestica* was more abundant in dairy cow barns than at other sites. In contrast, the number of *Musca sorbens* was greater at defecation grounds and in market areas [27]. This might be related to preference differences for breeding places and food sources in different fly populations.

**Table 2:** **Natural occurrence of *Cryptosporidium *** **and *Giardia *** **in flies worldwide**

**Country**	**Method**	***Cryptosporidium *** **(%)**	***Giardia *** **(%)**	**Ref**
Ethiopia	Modified Ziel-Neelson staining	*Cryptosporidium* (unspecified)	*G. duedenalis* (unspecified)	[25]
	Acid-fast staining	*Cryptosporidium* (69.2)		[27]
	Modified Ziel-Neelson staining	*Cryptosporidium* (16.7)	*G. duedenalis* (10.4)	[26]
Nigeria	ModifiedZiel-Neelson staining	*Cryptosporidium* (1.81)	*G. duedenalis* (3.34)	[28]
Poland	IFA and FISH	*C. parvum* (81)	*G. duedenalis* (84)	[29]
	Ziel-Neelson staining and PCR	*C. parvum* (18.9)		[30]
Spain	PCR	*C. parvum* (18)		[31]
	PCR		*G. duedenalis* (22)	[32]
USA	IFA	*C. parvum* (unspecified)		[33]
	PCR	*C. parvum* (83.3)		[34]
	FISH and FITC-conjugated MAb	*C. parvum* (80)	*G. duedenalis* (69)	[23]
	IFA and FISH	*C. parvum* (55.56)	*G. duedenalis* (7.94)	[35]

In the present study, the subtype IIdA19G1 identified in flies was identical to the cattle-derived isolates from the same dairy farms investigated [43]. Although the normal PCR used in the present study did not assess the viability of the oocysts in the flies, many previous studies have reported that viable *C. parvum* oocysts are present on the body surfaces/exoskeletons and in the digestive tracts of flies [23,29,35]. Thus, flies undoubtedly increase the risk to humans of infection with *Cryptosporidium* spp. and the cross-transmission to susceptible animal hosts, no matter where they occur in the flies. The molecular results described above not only provide powerful evidence of the mechanical transmission of these parasites by flies, acting as the transport hosts of *Cryptosporidium* in the dissemination of human cryptosporidiosis, but also allow us to assess the potential zoonotic transmission of cryptosporidiosis. *Cryptosporidium parvum* isolates from flies in the areas investigated were genetically identical to the human-derived *C. parvum* isolates from other countries at both the SSU rRNA locus and the *gp60* locus [44-48], implying its potential zoonotic transmission and its significance for public health. Because there is a lack of epidemiological data about human cases of cryptosporidiosis in the investigated areas, it is unclear whether the disease burden of human cryptosporidiosis is attributable to parasites of animal origin, and especially whether filth flies are involved in its mechanical transport. More systematic and complete epidemiological data are required from humans and animals in the areas investigated.

Although the three assemblage E isolates identified at the *tpi* locus had 100% similarity to those from cattle, sheep, and goats [49-52], new subtypes (different to isolates from other countries or areas) were detected at the *gdh* and *bg* loci, and shared 100% homology to those obtained from calves in the two dairy cattle farms investigated (data for cattle-derived *G. duodenalis* isolates unpublished). Thus, these findings demonstrate not only the natural occurrence of flies contaminated with *G. duodenalis* cysts, but also the endemic genetic characterization of *G. duodenali*s assemblage E in the dairy cattle in Henan, China. Previously, numerous molecular epidemiological data have shown the strong host specificity and narrow host range of assemblage E, which is predominantly found in cloven-hoofed domestic mammals, including cattle, water buffaloes, sheep, and pigs [17]. Flies contaminated with assemblage E *G. duodenalis* could increase the possibility of repeated infection or cross-transmission between these susceptible animals by mechanical transmission. However, the finding of flies carrying assemblage E does not pose a serious threat to the local inhabitants because assemblage E has currently only been isolated from three Egyptians [53]. In the present study, we detected no assemblage A or B *G. duodenalis* in flies, which are considered to be the two major assemblages causing human giardiasis. These results might reflect the lack or low frequencies of assemblages A and B in cattle, and may also be related to the relatively small numbers of infected flies detected. This study is the first molecular description of *G. duodenalis* isolates in flies at the genotype and subtype levels based on the *tpi*, *gdh*, and *bg* genes. Until now, in assessing the role of flies as vectors for the mechanical transmission of human giardiasis, only four other studies have identified *G. duodenali*s isolates in flies with molecular techniques, but the isolates were not genotyped [23,29,32,35].

## Conclusion

The occurrence of *Cryptosporidium* and *Giardia* in flies (*Musca domestica* is the major species) in China was first confirmed by molecular methods at both the genotype and subtype levels. The SSU rRNA and *gp60* sequences of *C. parvum* identified in flies were identical with animal and human isolates indicating that flies can act as an epidemiological link in zoonotic cryptosporidiosis. The variable PCR efficiencies at different loci of *Giardia* imply that we should use the MLG tool in future studies, because it could not only trace the infection or contamination source of *G. duodenalis* but also increase the detection rate. Although no disease outbreaks caused by *Cryptosporidium* or *Giardia* have been related to flies, flies carrying one or other of these parasites will significantly affect human health because of the huge numbers of flies in the environment. The proportions of human cryptosporidiosis and giardiasis that can be attributed to zoonotic transmission involving flies must be assessed using systematic molecular epidemiological data from humans and animals in these areas in the future.

## Competing interests

The authors declare that they have no competing interests.

## Authors’ contributions

LXZ, AQL, and FCJ conceived and designed the experiments; ZFZ, HJD, MQ, JFZ, and HYW performed the experiments; RJW, WZ, SMZ, and GYC analyzed the data; AQL, LXZ, and RJW wrote the manuscript. All the authors have read and approved the final manuscript.
